# Origin of White Electroluminescence in Graphene Quantum Dots Embedded Host/Guest Polymer Light Emitting Diodes

**DOI:** 10.1038/srep11032

**Published:** 2015-06-11

**Authors:** Jung Kyu Kim, Sukang Bae, Yeonjin Yi, Myung Jin Park, Sang Jin Kim, NoSoung Myoung, Chang-Lyoul Lee, Byung Hee Hong, Jong Hyeok Park

**Affiliations:** 1 SKKU Advanced Institute of Nanotechnology (SAINT) and School of Chemical Engineering and The Institute of Science and Technology, Sungkyunkwan University, 2066, Seobu-ro, Jangan-gu, Suwon 440-746, Republic of Korea; 2 Institute of Advanced Composite materials, Korea Institute of Science and Technology, San 101, Eunha-ri, Bongdong-eup, Wanju-gun, Jeollabuk-do, 565-905, Republic of Korea; 3 Department of Physics, Yonsei University, 50 Yonsei-ro, Seodaemun-gu, Seoul 120-749, Republic of Korea; 4 Department of Chemistry, Seoul University, Seoul 151-747, Republic of Korea; 5 Advanced Photonics Research Institute (APRI), Gwangju Institute of Science and Technology (GIST), Gwangju 500-712, Republic of Korea; 6 Department of Chemical and Biomolecular Engineering, Yonsei University, 50 Yonsei-ro, Seodaemun-gu, Seoul 120-749, Republic of Korea

## Abstract

Polymer light emitting diodes (PLEDs) using quantum dots (QDs) as emissive materials have received much attention as promising components for next-generation displays. Despite their outstanding properties, toxic and hazardous nature of QDs is a serious impediment to their use in future eco-friendly opto-electronic device applications. Owing to the desires to develop new types of nano-material without health and environmental effects but with strong opto-electrical properties similar to QDs, graphene quantum dots (GQDs) have attracted great interest as promising luminophores. However, the origin of electroluminescence from GQDs incorporated PLEDs is unclear. Herein, we synthesized graphene oxide quantum dots (GOQDs) using a modified hydrothermal deoxidization method and characterized the PLED performance using GOQDs blended poly(N-vinyl carbazole) (PVK) as emissive layer. Simple device structure was used to reveal the origin of EL by excluding the contribution of and contamination from other layers. The energy transfer and interaction between the PVK host and GOQDs guest were investigated using steady-state PL, time-correlated single photon counting (TCSPC) and density functional theory (DFT) calculations. Experiments revealed that white EL emission from the PLED originated from the hybridized GOQD-PVK complex emission with the contributions from the individual GOQDs and PVK emissions.

Electroluminescence from colloidal quantum dots (QDs), which was first reported in 1994, attracted lots of attentions from researchers working in the light-emitting diode (LED) fields. Since then, inorganic semiconductor quantum dots (QDs), such as CdSe, CdTe and PbTe have been extensively used as promising emissive materials for next-generation and low-cost LEDs, owing to their unique and attractive characteristics such as band-gap energies and narrow emission bandwidths that are easily tuneable by controlling the size of QDs during synthesis^1^,^2^,^3^,^4^,^5^,^6^. However, despite their outstanding characteristics, severe toxic and detrimental effects on humans are still crucial impediments to using inorganic QDs as next-generation luminophores in future opto-electronic device applications^7^,^8^.

Recently, zero-dimensional graphene quantum dots (GQDs) have attracted great interest as promising carbon based nanomaterials for opto-electronic devices^7^,^8^ and energy conversion systems^9^ owing to their unique properties: optical properties, which are easily tuneable in a sophisticated manner, substantial stability and good dispersity in chemicals, in addition to environmentally friendly properties. Unlike two-dimensional graphene sheets, GQDs can exhibit photoluminescence (PL) analogous to conventional inorganic QDs due to quantum confinement and edge effects^10^. Several research groups have demonstrated the possibility of controlling the band gap and PL emission of GQDs using chemical functionalization method^11^. Recently, organic and polymeric LEDs using GQDs as emissive materials have been reported. Rhee’s group reported electroluminescence (EL) from OLEDs which use GQDs embedded a 4,4′-bis(carbazol-9-yl)biphenyl (CBP) layer as emissive layer^12^. Here, GQDs were synthesized by amidative cutting of tattered graphite (Ami-GQD). Jeon’s group also reported similar results using poly(9-vinylcarbazole (PVK) as host matrix^13^. In both cases, it is suspected that EL from GQDs originated from (1) electron and hole transfer from the host to GQDs, which were injected into the host from the electron and hole transporting layers; (2) direct electron and hole injection into GQDs from the electron and hole transporting layers; and (3) energy transfer from the host to GQDs guest. However, a clear physical mechanism underlying the EL emission from GQDs in the device or the origin of the EL emission has not been demonstrated. In addition, reasons for the broadening of EL spectrum and white EL emission from the GQDs embedded PLED^12^, even though GQDs originally exhibited a greenish blue PL emission in the solution, were not clearly revealed.

In this study, we synthesized chemically functionalized fluorescent graphene oxide quantum dots (GOQDs) using a modified hydrothermal deoxidization method and fabricated simple structure of a PLED by blending GOQDs into a poly(N-vinyl carbazole) (PVK) polymer matrix. The PLED exhibited white EL with maximum peak at ~500 nm. The origin of white EL emission from the PLED was investigated using steady-state PL, time-correlated single photon counting (TCSCP) and density functional theory (DFT) calculations. The results indicated that white EL emission from the PLED originated from the hybridized GOQDs-PVK complex emission with the contributions from individual GOQDs and PVK emissions.

## Results

### Synthesis and characterization of GOQDs

GOQDs were synthesized by modified hydrothermal deoxidization cutting of graphene oxide sheets (GOSs), which were prepared from natural graphite powder using a modified Hummers method^14^. GOSs were chemically cut by oxidation in a solution mixture of H_2_SO_4_/HNO_3_ after thermal reduction at 250 °C for 2 h^10^. During the oxidation, epoxy groups were formed on GOSs through the epoxy chains, and groups could be easily converted into more energetically stable carbonyl groups by further oxidation, resulting in the cutting of sheets. GOQDs were synthesized by mild sonication of oxidized GOSs for 24 h, filtered with a 20 nm nano-porous Anodisc filter, followed by further filtration using a 3500Da dialysis bag.

**Figure 1:**
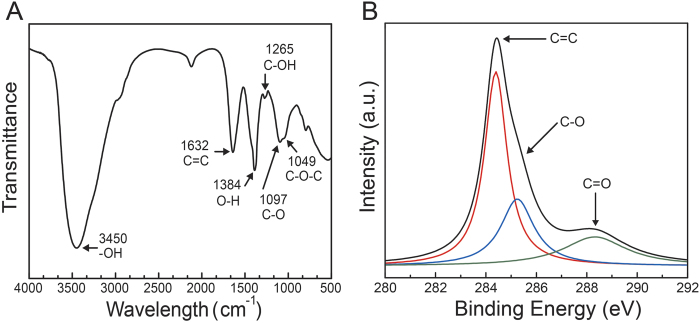
FT-IR and XPS. (**A**) Fourier-transform infra-red (FT-IR) spectra and (**B**) XPS C1s spectra of GOQDs.

**Figure 2:**
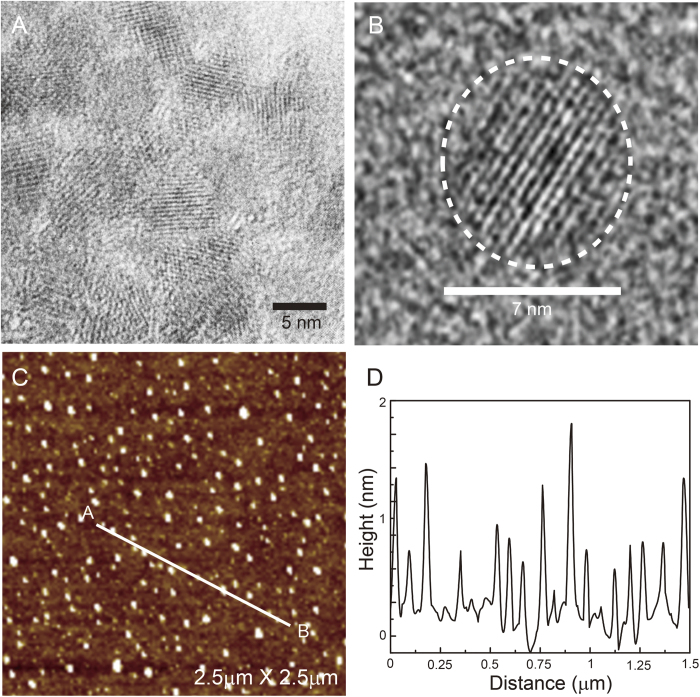
HR-TEM and AFM. High-resolution transmission electron microscopy images (**A, B**). AFM images of GOQDs on SiO_2_/Si substrate (**C**) and height distribution from A to B (**D**).

As observed in the Fourier transform infra-red (FT-IR) spectra (Fig. 1A), GOQDs exhibit an -OH peak at 3450 cm^−1^, a C = C stretching peak at 1632 cm^−1^, an O-H stretching peak at 1384 cm^−1^, a C-OH stretching peak at 1265 cm^−1^, a C-O stretching peak at 1097 cm^−1^ and a C-O-C stretching peak at 1049 cm^−1^. X-ray photoelectron spectroscopy (XPS) was performed to determine the composition of GOQDs (Fig. 1B). The XPS spectra contain a dominant SP^2^ C1s peak (C = C) at 284.7 eV, a C-O peak at 286.4 eV and a C = O peak at 288.2 eV. The presence of C = C, C-O and C = O bands indicated that GOQDs were functionalized with carboxyl and carbonyl groups. Owing to hydrothermal deoxidization, the oxygen-related moieties were located at the edge of GOQDs, resulting in capping conjugated SP^2^ carbons with SP^3^ carbons^15^. Figure 2 presents high-resolution transmission electron microscopy (HR-TEM) and atomic force microscopy (AFM) images of GOQDs. GOQDs are uniformly distributed without aggregation, and the size of GOQDs was ~7 nm, as determined from HR-TEM analysis. The height of GOQDs on SiO_2_/Si substrates was ~1.5 nm based on the height line profile, which indicates that GOQDs consisted of approximately 2–3 layers.

### Optical properties of GOQDs

**Figure 3:**
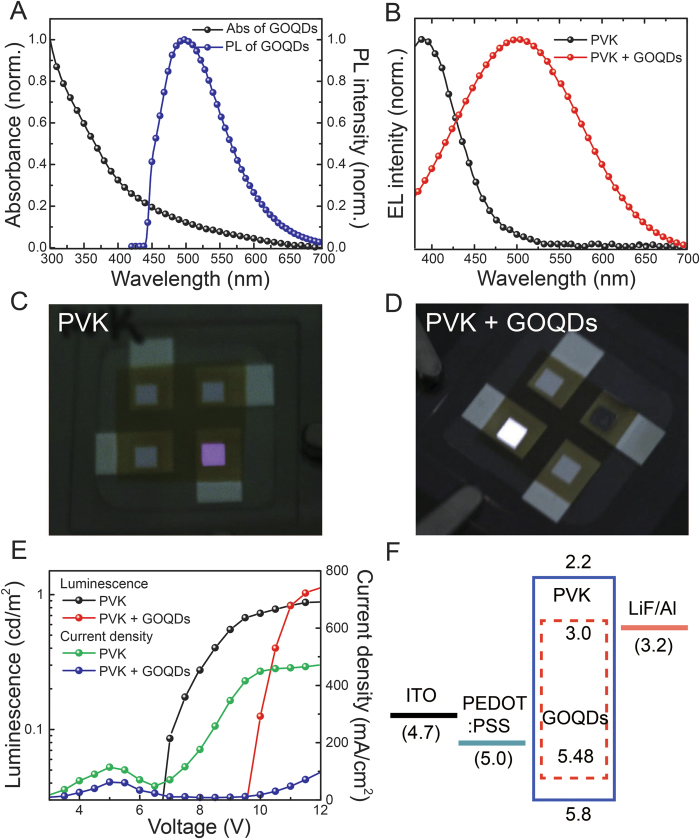
Device performance and characterization. (**A**) UV-visible absorption and PL spectrum of GOQDs. EL spectra (**B**) and digital camera images (**C** and **D**) of PLEDs using pristine PVK and GOQDs blended PVK as emissive layers, respectively (applied bias was 11 V). Current density and luminance vs. voltage curves (**E**) and schematic energy diagram (**F**) of PLEDs.

The optical properties of GOQDs were investigated using UV-Vis absorbance, PL and PL excitation (PLE) measurements. Figure 3A presents the UV-visible absorption and PL spectra of GOQDs, well dispersed in chlorobenzene solvent (4 mg/ml). GOQDs exhibited typical strong absorption in the UV region and the spectrum was extended up to 700 nm with a long-tail. The long-tail absorption strongly indicated the existence of defect sites in GOQDs^16^. From the PLE result, it is confirmed that the absorption peak in the UV region (below 300 nm) originated from the π → π^*^ transition of aromatic C = C bonds^17^ and at longer than 300 nm was assigned to the *n* → π^*^ transition of the substituent, which contained the non-bonding electrons (C = O bonds) (Figure S1 in supplementary information)^15^. The monochromatic excitation source (λ_exc_ = 350 nm) was used to measure the PL spectra of GOQDs. When its size is less than 100 nm, graphene can emit strong PL due to quantum confinement^18^,^19^,^20^. Although exact PL mechanisms of GOQDs are still under debated, many results suggest that the PL of GOQDs is attributed to two main processes: intrinsic state emission and defect state emission^21^. The former is induced by either the quantum size effect^22^, zigzag edge sites^10^,^23^ or the recombination of localized electron-hole pairs^24^,^25^, and the latter arises from defect sites^22^,^26^,^27^, such as vacancies, interstitial atoms, various functional groups in the graphene surface and edges. The PLE spectrum of GOQDs explained the origin of PL in GOQDs. GOQDs exhibited two PLE peaks at ~267 nm and ~325 nm. The PLE peak in the higher energy region originated from the intrinsic state, and the PLE peak in the lower energy region was attributed to the oxygen-related functional groups in GOQDs^28^. The relative PLE intensity at ~267 nm and ~325 nm was almost 1:1. Therefore, from the PLE result, it is suggested that both the intrinsic state and surface defect state originated from the oxygen-related functional groups at the graphene surface and edges almost equally contributed to the PL of GOQDs.

To investigate quantitatively relative contribution of the intrinsic state and oxygen-based functional groups to the PL emission as well as exciton lifetime of GOQDs, the PL decay profile of GOQDs (Figure S2 in supplementary information) was measured using time-correlated single photon counting (TCSPC). The PL decay profile, expressed by a bi-exponential fitting, indicated that the PL decay occurred through two relaxation pathways. Table S1 in supplementary information summarized PL lifetimes and fractional intensities of GOQDs. It is reported that the PL emission originated from the defect state generally exhibited a longer exciton lifetime than that from the intrinsic state^26^,^27^. The GOQDs exhibited two exciton lifetimes of 2.08 ns (50%) and 0.71 ns (50%). These results indicated that the luminescence of GOQDs equally contributed by intrinsic state and oxygenous functional groups, which corresponds well with the PLE result.

### EL from GOQDs blended PVK LED

Polymer light-emitting device using GOQDs blended PVK as emissive layer was fabricated and characterized. PVK was used as the polymer matrix due to its good hole transporting and optical properties, as well as favourable film forming properties^29^. The fabricated PLED device had a simple structure of ITO/PEDOT:PSS (40 nm)/emitting layer (80 nm)/LiF (1 nm)/Al (100 nm). The PEDOT:PSS and LiF were used as hole and electron injecting layers, respectively. In this experiment, simple device structure was selected to exclude the contribution of and contamination from other layers (e.g., electron transporting and hole blocking layers) to the EL spectrum and device performance. In particular, 2-(4-biphenylyl)-5-(4-tert-butylphenyl)−1,3,4-oxadiazole (PBD) or (1,3,5-tri(phenyl-2-benzimidazolyl)-benzene) (TPBI), commonly used as electron transporting layers for multi-layer organic or polymer light emitting diodes, have their own EL in the 400 nm to 500 nm region, resulting in bluish-white EL when they are combined with polymer matrixes^30^,^31^,^32^. Therefore, to more clearly understand the origin of EL emission from GOQDs in our host/guest system, all of the contaminating factors must be excluded from the device.

The PLED showed white EL emission with a maximum peak at ~500 nm (Fig. 3B). The EL spectrum was more broaden than PL spectrum of GOQDs in solution. However, the EL peak position was same as that of PL maximum of GOQDs. Therefore, it is supposed that the EL maximum peak at ~500 nm originated from GOQDs. Figure 3C,D present digital camera images of the EL emissions from PLEDs consisting of pristine PVK (violet emission) and GOQDs blended PVK (white emission) as emissive layers, respectively. The reason for the broadness of EL spectrum as well as white emission will be discussed in the density functional theory (DFT) calculations section. Current density and luminescence vs. voltage curves of PLEDs are displayed in Fig. 3E. The PLED using GOQDs blended PVK as emissive layer exhibited a lower current density than that of the PLED using PVK as emissive layer owing to better charge balance achieved by GOQDs. The device performance of our simple structured PLED is relatively poor (maximum luminance is 1 cd/m^2^ at 11 V) compared with reported results^13^. However, this simple structure of PLED is more suitable to reveal the origin of EL emission in PLEDs, which satisfies our research purpose.

**Figure 4:**
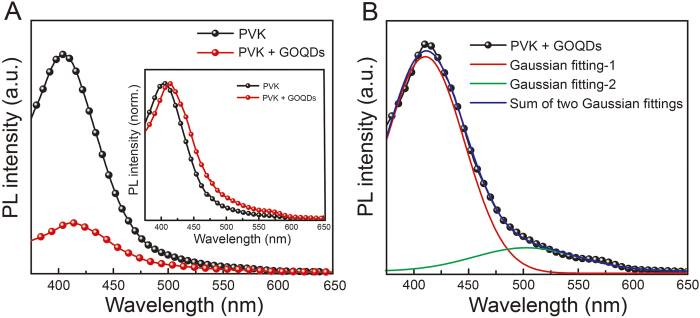
Photoluminescence. (**A**) PL spectra of pristine PVK and GOQDs blended PVK films. (**B**) Double Gaussian fitting of PL spectra of GOQDs blended PVK film. Insert in (**A**): Normalized PL spectra of pristine PVK and GOQDs blended PVK films.

It is suggested that the EL emission of GOQDs in PLED is attributed to three mechanisms: (1) electron and hole transfer from the host to GOQDs, which were injected into the host from electron and hole transporting layers, (2) energy transfer from the PVK host to GOQDs guest; and (3) direct charge injection to GOQDs. Here, the mechanism (1) was not considered because its probability was very low. For efficient Förster energy transfer from the host to guest, the absorption of the guest material must spectrally overlap with the PL emission of the host^33^. From UV-Vis absorption of GOQDs and the PL spectrum of PVK, it is evident that prerequisite conditions for Förster energy transfer are satisfied. Figure 4A presents the PL spectra of pristine PVK and GOQDs blended PVK films. The excitation wavelength for the PL measurements was 350 nm. Significant PL quenching of PVK emission at ~405 nm, originated from carbazole excimer, is observed in the blended film. The PL quenching ratio of carbazole excimer emission reached almost 80%. To satisfy energy transfer, the guest emission (GOQDs) must appear in the PL spectrum with a corresponding decrease of the host PL emission. Owing to low PL emission intensity of GOQDs, the presence of PL emission of GOQDs was monitored after normalization of the PL spectra of pristine PVK and GOQDs blended PVK films (Fig. 4A, **inset)**. A slight red shift of the PL spectrum and higher PL intensity at entire wavelength were observed compared with those of pristine PVK film. The reason for these phenomena will be discussed in the DFT calculations section. Figure 4B shows double Gaussian fitting of the PL spectra of the GOQDs blended PVK film. In contrast to the PL spectrum of pristine PVK, which consisted of a single Gaussian curve, the PL spectrum of the GOQDs blended PVK film can be divided into two PL spectra, originated from the PVK and GOQDs moieties. Based on this result, it is suggested that energy transfer between the PVK host and GOQDs guest occurred.

**Figure 5:**
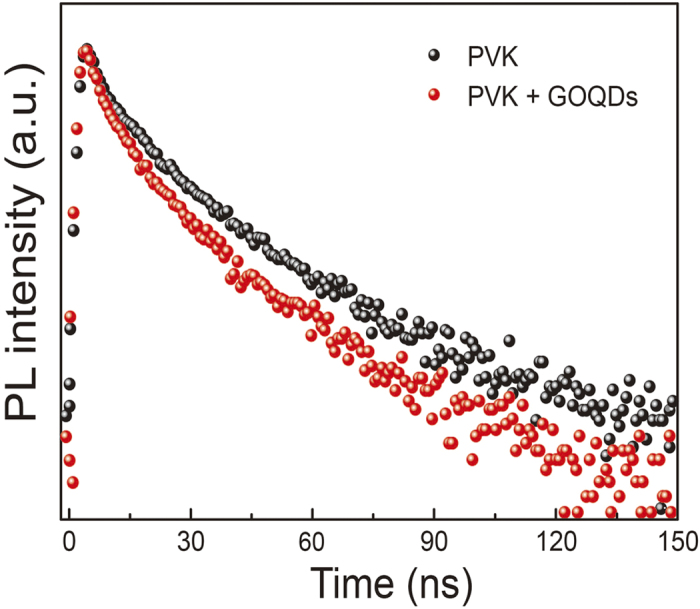
Time-resolve PL. PL decay profiles of pristine PVK and GOQDs blended PVK films.

To quantitatively evaluate the energy transfer efficiency between PVK host and GOQDs guest, a TCSPC experiment was conducted. Figure 5 presents the PL decay profiles of pristine PVK and GOQDs blended PVK films. The decay profiles were fitted using bi-exponential fitting. Table 1 summarized the PL lifetimes and fractional intensities of pristine PVK and GOQDs blended PVK films. The amplitude-weighted average lifetime (τ_ave_) of PVK decreased from 10.36 to 8.11 ns when GOQDs were blended in the PVK, which is consistent with steady-state PL result. In more detail, τ_1_ decreased from 4.63 to 4.23 ns, and τ_2_ decreased from 19.71 to 16.73 ns. The decrease ratio of τ_2_ is larger than that of τ_1_, which suggests that the carbazole excimer more contributed to the energy transfer process than that of isolated carbazole moiety^34^.

The energy transfer efficiency was calculated using the following equation^35^:


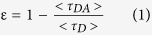


where ε = 0.22 when the amplitude-weighted average lifetime was used. Based on this result, the occurrence of energy transfer between PVK and GOQDs was confirmed.

The probability of direct charge injection to GOQDs was also investigated by analyzing the energy levels of PVK and GOQDs. Figure 3F shows the energy levels of PVK and GOQDs. The HOMO and LUMO energy levels of PVK obtained from the literature are 5.4–5.8 eV and 1.9–2.2 eV, respectively^29^. The HOMO and LUMO energy levels of GOQDs were 5.48 and 3.0 eV^15^,^36^, respectively, determined from ultraviolet photoemission spectroscopy (UPS) (Figure S3 in supplementary information) and the optical band gap, which was estimated by absorption and PL results. As observed in the energy diagram in Fig. 3F, the HOMO and LUMO energy levels of GOQDs are located within those of the PVK, and have a small energy barrier for charge injection from both electrodes; therefore, direct charge injection is possible in this system^36^. Thus, the EL emission of GOQDs could have originated from both energy transfer from PVK host and direct charge injection to GOQDs.

### DFT calculations and origin of white EL emission in PLED

Based on experimental results from the steady-state PL, TCSPC and analysis of energy levels, it was confirmed that the EL emission at ~500 nm originated from GOQDs. However, unsolved questions remained: What are the reasons for (1) broadening of EL spectrum and white emission, (2) slight red shift of the PL spectrum and higher PL intensity at the entire wavelength in GOQDs blended PVK film? To determine these reasons, DFT calculations which can uncover the interaction between PVK and GOQDs were performed. The GOQD was modelled with a 19-ring graphene structure with hydrogen termination at the edges for proper relaxation. The unit structure of PVK was used because the side chain does not contribute to frontier orbitals, which are associated with essential interactions^37^.

**Figure 6:**
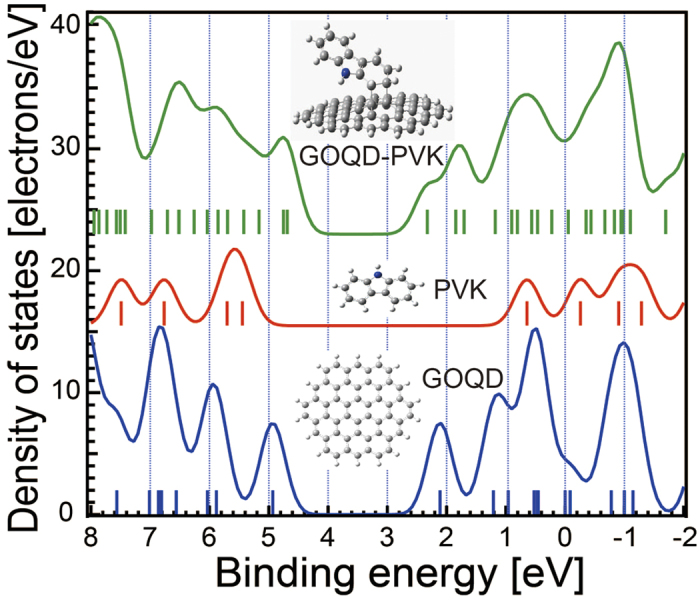
DFT calculations. Simple simulation using DFT for the calculation of molecular orbital (MO) levels and density of states (DOS) of pristine GOQD, PVK and GOQD-PVK complex. The DOS of pristine GOQD (blue line), pristine PVK (red line) and GOQD-PVK (green line) are shown. The binding energy of each MO is indicated with vertical bars. The insert image shows the most stable formation of pristine GOQD, PVK and GOQD-PVK with this calculation.

The molecular orbital (MO) energy and simulated density of states (DOS) are shown in Fig. 6. Four possible initial geometries of GOQD-PVK systems were designed according to their molecular symmetry, and each system was fully relaxed to find the energetic minima. The minimum energy geometry is shown in Fig. 6 **(top inset)**. The other structures obtained from DFT calculations have far higher total energy compared with the thermal energy of room temperature. As seen in the topmost DOS in Fig. 6, there are significant MO hybridizations between GOQD and PVK because the GOQD-PVK DOS cannot be simply deconvoluted into each PVK and GOQD DOS. This finding implies that there are significant interactions between GOQD and PVK, which could affect PL or EL emissions.

**Figure 7:**
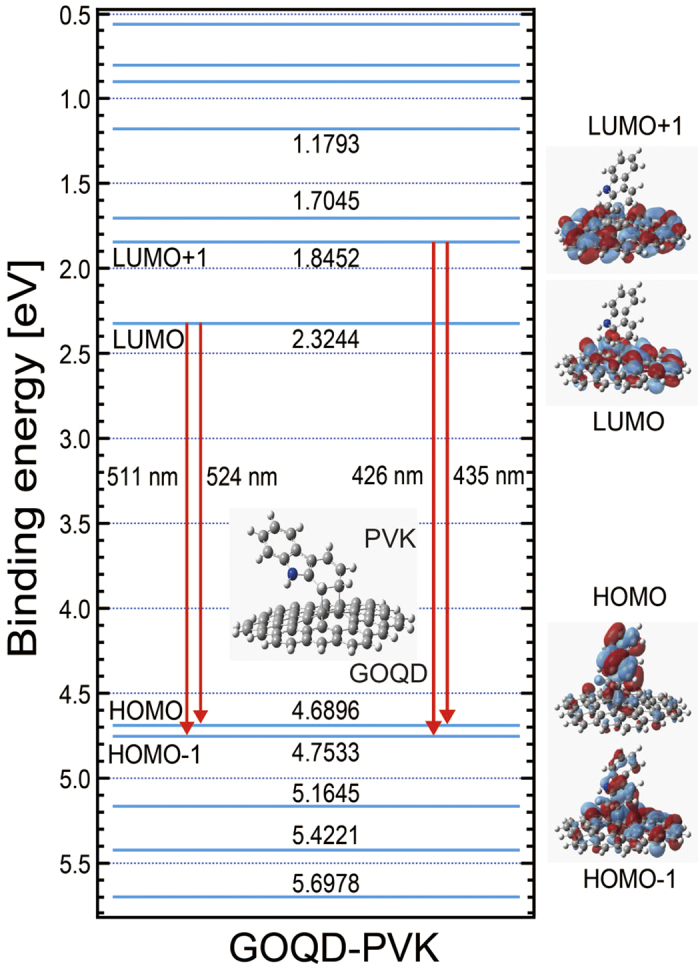
Calculated MO and electric transition. The MO and possible electronic transition associated with PL and EL. Wave functions of selected MO are also presented.

The electronic transition related to PL or EL emissions can be estimated with MO structures as a first approximation within the framework of Koopmans’ theorem^38^. To see detailed transition, MOs of the GOQD-PVK complex system are presented in Fig. 7. Nearly degenerated HOMO and HOMO−1 levels are located at 4.69 and 4.75 eV, while the LUMO and LUMO + 1 levels are located at 2.32 and 1.85 eV, respectively. Possible electronic transitions for PL or EL emissions are indicated with red arrows. The emission features measured near ~500 nm correspond to the transition from the LUMO to the nearly degenerated HOMO states (511 and 524 nm). The other features measured near ~410 nm are associated with the transition from LUMO+1 to degenerated HOMO (426 and 435 nm). These two main emission features are quite close to those of individual GOQDs and PVK, as observed in the PL emission (Fig. 3). However, these two emission features from the hybridized GOQD-PVK complex have completely different origins: these features are associated with the HOMO and HOMO−1, where their wave functions are highly delocalized over both moieties of GOQD and PVK, while LUMO and LUMO + 1 are associated with a relatively localized GOQD moiety (wave functions are indicated on the right side). This finding means that PL and EL emissions (Fig. 3B and Fig. 4) are not solely related to individual GOQDs or PVK but also related to the hybridized GOQD-PVK complex states.

We assume that white EL emission from the GQDs incorporated PLEDs will occur when organic or polymeric materials, which have carbazole moieties, were used as host. In previous result^12^, white EL emission was also observed when the CBP was used as host material. To reveal our hypothesis that carbazole moieties have a crucial role to generate white EL emission, DFT calculations (calculated MO and electric transition) of GOQD-CBP was conducted (Figure S4 in supplementary information). Analogous to GOQD-PVK system, two emissions at ~513 and ~433 nm were observed, which originated from the hybridized GOQD-CBP complex states. From this result, it is confirmed that host materials containing carbazole moieties can make hybridization complex states with GQDs and this hybridization has a crucial role to generate white EL emission.

## Conclusion

We synthesized chemically functionalized fluorescent GOQDs using a modified hydrothermal deoxidization method and fabricated a simple structured PLED by blending GOQDs into a PVK polymer host matrix. The basic device structure was used to reveal the origin of EL by excluding the contribution of and contamination from other layers. The PLED exhibited white EL emission with a maximum peak at ~500 nm. To evaluate quantitatively the energy transfer efficiency between PVK host and GOQDs guest, a TCSPC experiment was conducted. The amplitude-weighted average lifetime (τ_ave_) of PVK decreased from 10.36 to 8.11 ns when GOQDs were blended in the PVK, which is consistent with steady-state PL result, thereby energy transfer between PVK and GOQDs was confirmed. Furthermore, from DFT calculations, it is concluded that (1) broadening of EL spectrum and white emission, (2) slight red shift of PL spectrum and higher PL intensity at the entire wavelength region in the GOQDs blended PVK film are caused by the contribution from the hybridized GOQD-PVK complex emission. Therefore, it was concluded that white EL emission in the PLED originated from the hybridized GOQD-PVK complex emission with contributions from individual GOQDs and PVK emissions. This work demonstrates strong possibility that GOQDs can be used as inexpensive and eco-friendly electronic materials for opto-electronic devices such as displays and lighting devices in the near future.

## Methods

### Synthesis of GOQDs

Graphene oxide (GO) sheets were synthesized from natural graphite powder using the modified Hummers method^14^. To remove any acid, GO was centrifuged (4000 rpm, 30 min) and then diluted with distilled water (DI water). Any remaining acid was further removed by rinsing, through centrifugation in water 6 times. To obtain small and uniform sized reduced graphene oxide (RGO) sheets, purified GO sheets were re-dispersed in DI water and then subsequently deoxidised in a box furnace at 250 °C for 2 h under ambient N_2_. Then, 0.5 g of reduced RGO sheets was oxidised in a mixture of sulphuric acid and nitric acid (100 : 300 ml) for 20 h under mild ultra-sonication. The mixture of acids was diluted *via* previously used rinsing process, and resulting oxidised graphene sheets were re-dispersed in DI water for 24 h under mild sonication. Then, smaller oxidised graphene sheets were filtered with a 0.02-μm nano-porous Anodisc filter. Finally, to obtain the GOQDs, the mixture was further filtered overnight using a 3500 Da dialysis bag.

### Fabrication of PLEDs

The fabricated PLED devices had a structure of ITO/ PEDOT:PSS(40 nm)/emitting layer(80 nm)/LiF(1 nm)/Al(100 nm). ITO glass (25 mm 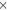 25 mm) was cleaned with organic solvents and UV-ozone for 20 min. The hole injection PEDOT:PSS (AI4083) layer was spin coated on the ITO with a thickness of 40 nm and dried at 150 °C for 15 min in an oven. Then, pristine PVK and a 0.4 wt % GOQDs blended PVK (Tokyo Chemical Industry) layers were spin coated on the PEDOT:PSS layer and used as emitting layers, respectively. Chlorobenzene was used as the solvent for dissolving GOQDs and PVK. After spin casting, the emitting layers with 80 nm thickness were treated at 80 °C for 30 min in an argon (Ar) filled glove box. At the base pressure of ~10^−7^ Torr, 1 nm thick lithium fluoride (LiF) and 100 nm thick aluminium (Al) layers were thermally evaporated on top of the emitting layers as a cathode. The size of emitting area was ~ 4 mm^2^.

### Characterization of synthesized GOQDs and PLEDs

The structural quality and crystallinity of GOQDs were inspected using high-resolution transmission electron microscopy (HR-TEM) (JEM-3010, JEOL Company). The thickness and uniformity of spin coated GOQDs on the SiO_2_ substrate were investigated using atomic force microscopy (AFM) (XE−100, Park system). X-ray photoelectron spectroscopy (XPS, Thermo Scientific K-Alpha small-spot system) and Fourier-transform infrared spectroscopy (FT-IR, Thermo Scientific Nicolet 6700 spectrometer) were employed to confirm functional groups of GOQDs. UPS measurement was performed in the homemade *in-situ* photoemission analysis system (generator: VUV 5000 and detector: SES-100) with a *hv* = 40.8 eV, He II source. In order to prepare the samples for XPS, FT-IR, and UPS measurements, we used exactly same manner as it was in our previous report.^15^ The UV-visible absorption spectrum was measured using a spectrophotometer (Shimadzu UV-2401PC), and PL spectra were measured using a 15 cm monochromator (SP-2150i, Acton) with photomultiplier tube (PMT) and tuneable Ti:sapphire laser (Mira900, Coherent) and/or Hitachi F-7000 spectrofluorometer equipped with a Xenon lamp as an excitation light. The current density and luminescence vs. voltage characteristics of the PLED were recorded using a source measurement unit (Keithley 2400) and a luminance meter (Minolta CS100). The PL decay of GOQDs in chlorobenzene solution was investigated using the time-correlated single photon counting (TCSPC) measurement. The second harmonic (SHG = 350 nm) of a tuneable Ti:sapphire laser (Mira900, Coherent) with a ∼150 fs pulse width and 76 MHz repetition rate was used as an excitation source. The PL emission was spectrally resolved using some collection optics and a monochromator (SP-2150i, Acton). The TCSPC module (PicoHarp, PicoQuant) with a MCP-PMT (R3809U-59, Hamamatsu) was used for ultrafast detection. The total instrument response function (IRF) for PL decay was less than 140 ps, and the temporal time resolution was less than 10 ps. The deconvolution of the actual fluorescence decay and IRF was performed using fitting software (FlouFit, PicoQuant) to deduce the time constant associated with each exponential decay. The PL decay of pristine PVK and GOQDs blended PVK films was also investigated using TCSPC measurement. The Chameleon laser system, which consists of a diode laser pumping a Ti:sapphire laser (Chameleon Ultra II, Coherent Inc.), was mode-locked to provide picosecond or femtosecond pulses of light with approximately ~150 fs pulse duration and an 80 MHz repetition rate. The laser was used in combination with a pulse picker (9200 series, Coherent Inc.), which was modified at 4.4 MHz pulse frequency, and a 2^nd^ harmonic generation signal from BaBO_4_ crystals (β-barium borate: BBO), which could frequency double the Chameleon’s output to create wavelengths of 350 nm. The fluorescence emission was filtered by an appropriate long pass filter (355 nm) to remove residual excitation light and focused into the entrance slit of a spectrometer (SP-2300i, Acton) with a spectral resolution of approximately 1 nm. The spectrometer was coupled to a picosecond streak camera system (C9300, Hamamatsu Photonics).

## Additional Information

**How to cite this article**: Kim, J. K. *et al.* Origin of White Electroluminescence in Graphene Quantum Dot Embedded Host/Guest Polymer Light Emitting Diodes. *Sci. Rep.* **5**, 11032; doi: 10.1038/srep11032 (2015).

## Supplementary Material

Supplementary Information

## Figures and Tables

**Table 1:** PL lifetime of pristine PVK and GOQDs blended PVK films^a^.

**Polymer films**	***τ***_**1**_(***f***_**1**_**)/ns**	***τ***_**2**_(***f***_**2**_**)/ns**	***τ***_**avr**_**/ns**^b^	**χ**^**2**^^c^
Pristine PVK	4.63 (0.62)	19.71 (0.38)	10.36	1.231
GOQDs blended PVK film	4.23 (0.69)	16.73 (0.31)	8.11	2.181

^a^The monitored wavelength was 410 nm. The PL decay curves were fitted to a bi-exponential function to calculate the lifetime of the samples.

^b^The amplitude-weighted average exciton lifetime (τ_avr_) was *f*_1_*τ*_1_ + *f*_2_*τ*_2_, where *f*_1_ and *f*_2_ are fractional intensities and *τ*_1_ and *τ*_2_ are lifetimes.

^c^χ^2^ is the reduced chi-square value.

## References

[b1] Murray C. B., Norris D. J. & Bawendi M. G. Synthesis and characterization of nearly monodisperse CDE (E=sulfur, selenium, tellurium) semiconductor nanocrystallites. *J. Am. Chem. Soc*. 115, 8706–8715 (1993).

[b2] Dabbousi B. O., Bawendi M. G., Onitsuka O. & Rubner M. F. Electroluminescence from CdSe quantum-dot/polymer composites. *Appl. Phys. Lett*. 66, 1316–1318 (1995).

[b3] Schlamp M. C., Peng X. & Alivisatos A. P. Improved efficiencies in light emitting diodes made with CdSe(CdS) core/shell type nanocrystals and a semiconducting polymer. *J. Appl. Phys*. 82, 5837–5842 (1997).

[b4] Colvin V. L., Schlamp M. C. & Alivisatos A. P. Light-emitting diodes made from cadmium selenide nanocrystals and a semiconducting polymer. Nature 370, 354–357 (1994).

[b5] Coe S., Woo W. K., Bawendi M. G. & Bulović V. Electroaluminescence from single monolayers of nanocrytals in molecular organic devices. Nature 420, 800–803 (2002). 12490945 10.1038/nature01217

[b6] Chaudhary S., Ozkan M. & Chan W. C. W. Trilayer hybrid polymer–quantum dot light-emitting diodes. *Appl. Phys. Lett*. 84, 2925–2927 (2004).

[b7] Bhattacharya P. & Mi Z. Quntum-dot optoelectronic devices, proceedings of the IEEE, 95, 1723–1740 (2007).

[b8] Valizadeh A. *et al.* Quntum dots: synthesis, bioapplications, and toxicity. Nanoscale research Lett. 7, 480 (2012). 10.1186/1556-276X-7-480 PMC3463453 22929008

[b9] Zhang Z., Zhang J., Chen N. & Qu L. Graphene quantum dots: an emerging material for energy-related applications and beyond. *Energy Environ. Sci*. 5, 8869–8890 (2012).

[b10] Pan D., Zhang J., Li Z. & Wu M. Hydrothermal route for cutting graphene sheets into blue-luminescent graphene quantum dots. *Adv. Mater*. 22, 734–738 (2010). 20217780 10.1002/adma.200902825

[b11] Tetsuka H. *et al.* Optical tunable amino-functionalized graphene quantum dots. *Adv. Mater*. 24, 5333–5338 (2012). 22833282 10.1002/adma.201201930

[b12] Kwon W. *et al.* Electroluminescence from graphene quantum dots prepared by amidative cutting of tatterd graphite. *Nano Lett*. 14, 1306–1311 (2014). 24490804 10.1021/nl404281h

[b13] Song S. H. *et al.* Highly Efficient Light-Emitting Diode of Graphene Quantum Dots Fabricated from Graphite Intercalation Compounds. *Adv. Optcal Mater*. 2, 1016–1023 (2014).

[b14] Hirata M., Gotou T., Horiuchi S., Fujiwara M. & Ohba M. Thin-film particles of graphite oxide 1: high-yield synthesis and flexibility of the particles. Carbon 42, 2929–2937 (2004).

[b15] Kim J. K. *et al.* Balancing light absorptivity and carrier conductivity of graphene quantum dots for high-efficiency bulk heterojunction solar cells. ACS Nano 7, 7207–7212 (2013). 23889189 10.1021/nn402606v

[b16] Liu F., Jang M.-H., Ha H. D., Kim J.-H., Cho Y.-H. & Seo T. S. Facile synthetic method for pristine graphene quantum dots and graphene oxide quantum dots: origin of blue and green luminescence. Adv. Mater. 25, 3657–3662 (2013). 23712762 10.1002/adma.201300233

[b17] Novoselov K. S. *et al.* Electric field effect in atomically thin carbon films. Science 306, 666–669 (2004). 15499015 10.1126/science.1102896

[b18] Ponomarenko L. A. Chaotic dirac billiard in graphene quantum dots. Science 320, 356–358 (2008). 18420930 10.1126/science.1154663

[b19] Li X., Wang X., Zhang L., Lee S. & Dai H. Chemically derived, ultrasmooth graphene nanoribbon semiconductors. Science 319, 1229–1232 (2008). 18218865 10.1126/science.1150878

[b20] Li Y. *et al.* An electrochemical avenue to green-luminescent graphene quantum dots as potential electron-acceptors for photovoltaics. Adv. Mater. 23, 776–780 (2011). 21287641 10.1002/adma.201003819

[b21] Zhu S. *et al.* Surface chemistry routes to modulate the photoluminescence of graphene quantum dots: from fluorescence mechanism to up-conversion bioimaging applications. Adv. Funct. Mater. 22, 4732–4740 (2012).

[b22] Zhu S. *et al.* Strongly green-photoluminescent graphene quantum dots for bioimaging applications. Chem. Commun. 47, 6858–6860 (2011). 10.1039/c1cc11122a 21584323

[b23] Pang J. *et al.* Graphene quantum dots derived from carbon fibers. Nano Lett. 12, 844–849 (2012). 22216895 10.1021/nl2038979

[b24] Eda G. *et al.* Blue photoluminescence from chemically derived graphene oxide. *Adv. Mater*. 22, 505–509 (2010). 20217743 10.1002/adma.200901996

[b25] Loh K. P., Bao Q., Eda G. & Chhowalla M. Graphene oxide as a chemically tunable platform for optical applications. Nat. Chem. 2, 1015–1024 (2010). 21107364 10.1038/nchem.907

[b26] Zhou X. *et al.* Photo-fenton reaction of graphene oxide: a new strategy to prepare graphene quantum dots for dna cleavage. ACS Nano 6, 6592–6599 (2012). 22813062 10.1021/nn301629v

[b27] Shen J., Zhu Y., Yang X., Zong J., Zhang J. & Li C. One-pot hydrothermal synthesis of graphene quantum dots surface-passivated by polyethylene glycol and their photoelectric conversion under near-infrared light. New J. Chem. 36, 97–101 (2012).

[b28] Petruska J. *et al.* Changes in the Electronic Transitions of Aromatic Hydrocarbons on Chemical Substitution. II. Application of Perturbation Theory to Substituted-Benzene Spectra. J. Chem. Phys. 34, 1120 (1961).

[b29] Kim J. K., Lee D.-H., Wang D. H., Cho S. M., Lee J. Y. & Park J. H. Efficient and low potential operative host/guest concentration graded bilayer polymer electrophosphorescence devices. J. Lumin. 132, 870–874 (2012).

[b30] Zhang C., von Seggern H., Kraabel B., Schmidt H.-W. & Heeger A. J. Blue emission from polymer light-emitting diodes using non-conjugated polymer blends with air-stable electrodes. Synth. Met. 72, 185–188 (1995).

[b31] Gao Z. Q. *et al.* Blue organic electroluminescence of 1,3,5-triaryl-2-pyrazoline. Synth. Met. 105, 141–144 (1999).

[b32] Wong T. C., Kovac J., Lee C. S., Hung L. S. & Lee S. T. Transient electroluminescence measurements on electron-mobility of N-arylbenzimidazoles. Chem. Phys. Lett. 334, 61–64 (2001).

[b33] Dogariu A., Gupta R., Heeger A. J. & Wang H. Time-resolved Förster energy transfer in polymer blends. Synth. Met. 100, 95–100 (1999).

[b34] Bonon B. M. A. & Atvars T. D. Z. Energy transfer from poly(vinyl carbazole) to a fluorene-vinylene copolymer in solution and in the solid state. Photochem. Photobiol. 88, 801–809 (2012). 22372524 10.1111/j.1751-1097.2012.01133.x

[b35] Bhattacharyya S., Sen T., & Patra A. Host−guest energy transfer: semiconducting polymer nanoparticles and au nanoparticles. J. Phys. Chem. C 114, 11787–17795 (2010).

[b36] Gupta V., Chaudhary N., Srivastava R., Sharma G. D., Bhardwaj R. & Chand S. Luminscent graphene quantum dots for organic photovoltaic devices. J. Am. Chem. Soc. 133, 9960–9963 (2011). 21650464 10.1021/ja2036749

[b37] Son D. I. *et al.* Bistable organic memory device with gold nanoparticles embedded in a conducting poly(n-vinylcarbazole) colloids hybrid. J. Phys. Chem. C 115, 2341–2348 (2011).

[b38] Koopmans T. Über die zuordnung von wellenfunktionen und eigenwerten zu den einzelnen elektronen eines atoms. Physica 1, 104–113 (1934).

