# Supplementary material for: Origin of White Electroluminescence in Graphene Quantum Dots Embedded Host/Guest Polymer Light Emitting Diodes
Source: Sci Rep. 2015 Jun 11;5:11032. doi: 10.1038/srep11032 (PMC4463941; doi:10.1038/srep11032)
Supplement: Supplementary Information [file srep11032-s1.doc]

Supplementary Information

Origin of White Electroluminescence in Graphene Quantum Dots Embedded Host/Guest Polymer Light Emitting Diodes

Jung Kyu Kim^1,6^, Sukang Bae^2^, Yeonjin Yi^3^, Myung Jin Park^4^, Sang Jin Kim^4^, NoSoung Myoung^5^, Chang-Lyoul Lee^5*^, Byung Hee Hong^4*^ and Jong Hyeok Park^6*^

^1^ SKKU Advanced Institute of Nanotechnology (SAINT) and School of Chemical Engineering and The Institute of Science and Technology, Sungkyunkwan University, 2066, Seobu-ro, Jangan-gu, Suwon 440-746, Republic of Korea

^2^ Institute of Advanced Composite materials, Korea Institute of Science and Technology, San 101, Eunha-ri, Bongdong-eup, Wanju-gun, Jeollabuk-do, 565-905, Republic of Korea

^3^ Department of Physics, Yonsei University, 50 Yonsei-ro, Seodaemun-gu, Seoul 120-749, Republic of Korea

^4^ Department of Chemistry, Seoul University, Seoul 151-747, Republic of Korea

^5^ Advanced Photonics Research Institute (APRI), Gwangju Institute of Science and Technology (GIST), Gwangju 500-712, Republic of Korea

^6^ Department of Chemical and Biomolecular Engineering, Yonsei University, 50 Yonsei-ro, Seodaemun-gu, Seoul 120-749, Republic of Korea

Correspondence and requests for materials should be addressed to C. L. L. (vsepr@gist.ac.kr), B. H. H. (byunghee@snu.ac.kr), J. H. P. (lutts@yonsei.ac.kr)

^*^ Corresponding authors: Dr. C.-L. Lee, Prof. B. H. Hong and Prof. J. H. Park


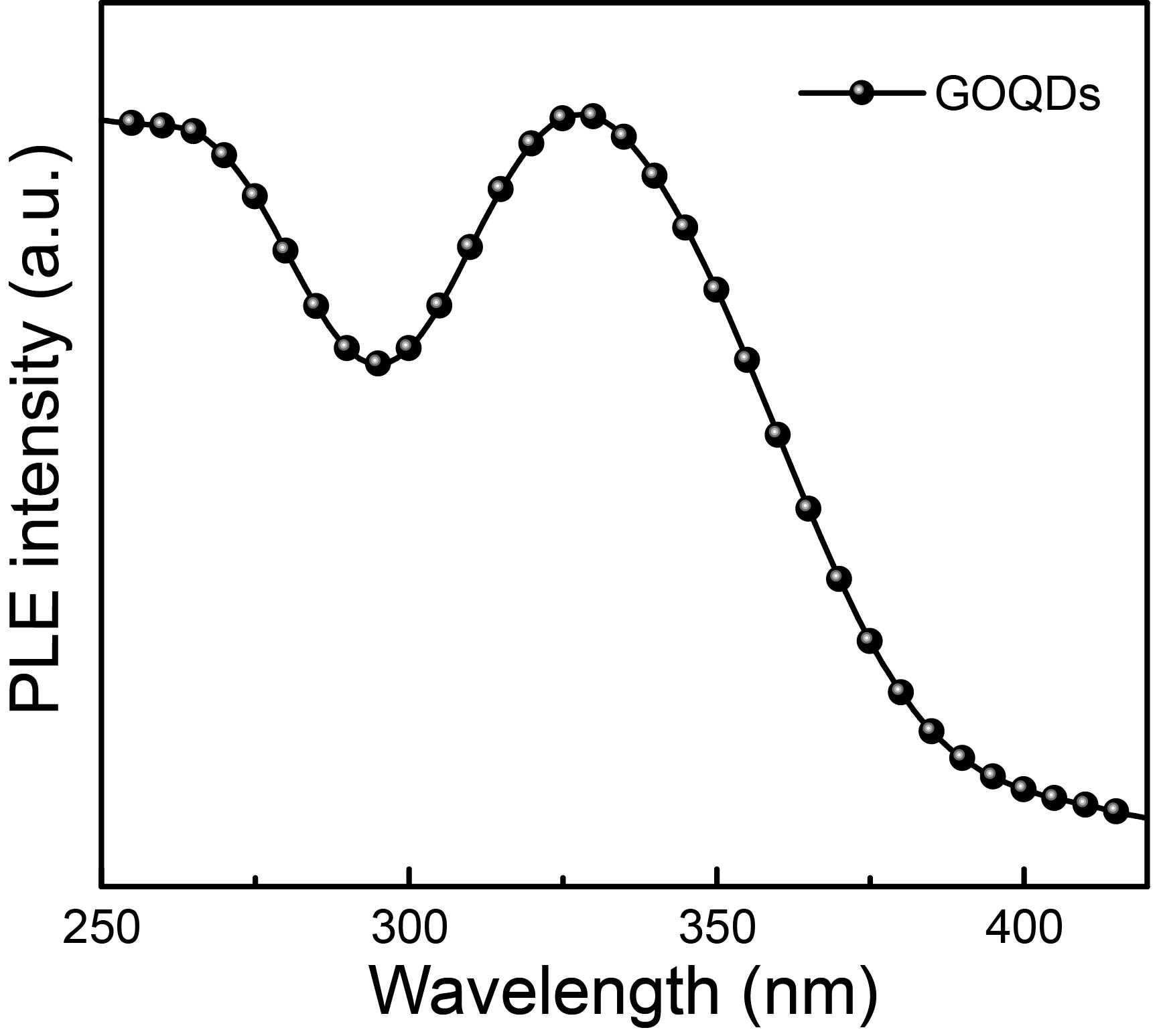


Figure S1. PLE spectrum of GOQDs in chlorobenzene solution.


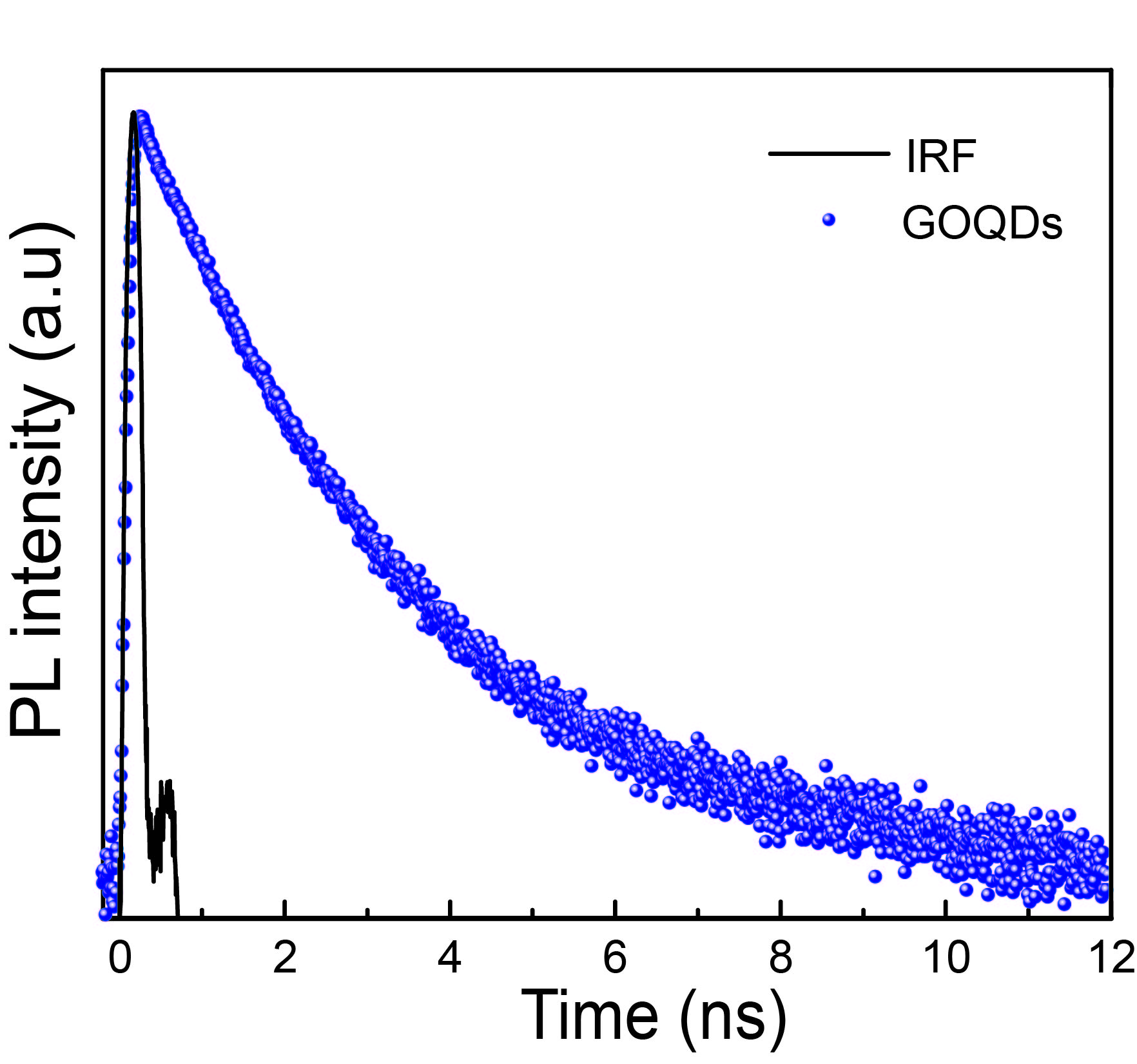


Figure S2. PL decay profile of GOQDs in chlorobenzene solution.


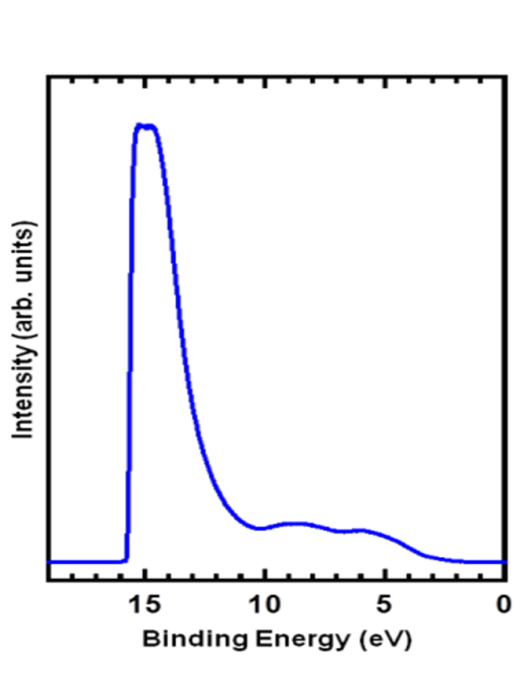


Figure S3. Ultraviolet photoemission spectroscopy (UPS) spectra of GOQDs. The UPS measurement were performed in a homemade *in situ* photoemission analysis system (generator: VUV 5000; detector: SES-100, *hν*=40.8 eV, He II source).


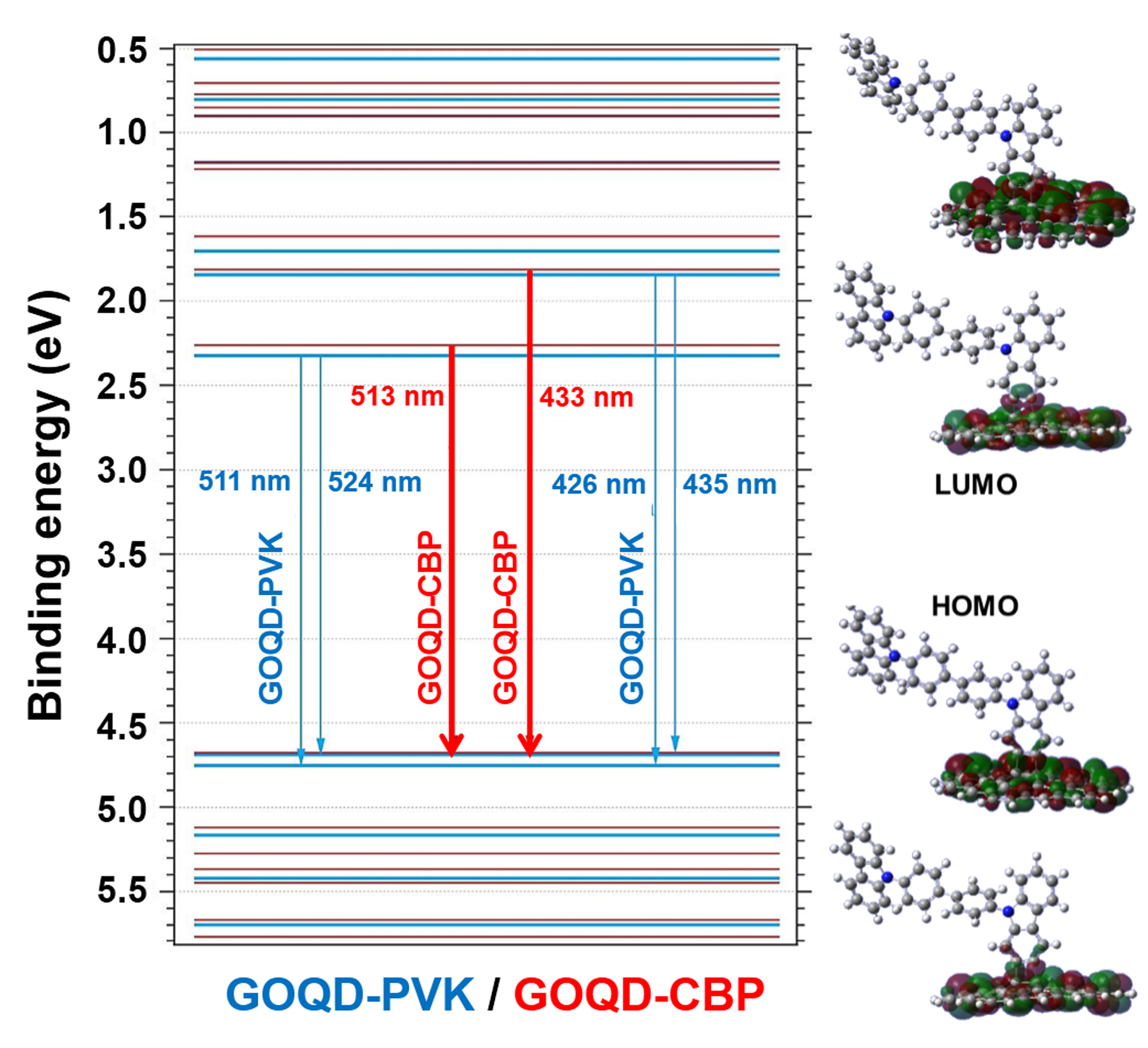


Figure S4. Calculated MO and electric transition of GOQD-CBP.

**Supplementary Table 1. PL lifetime of GOQDs in chlorobenzene solution^a^**.

| Materials | *τ*_1_(*f*_1_)/ns | *τ*_2_(*f*_2_)/ns | *τ*_avr_/ns^b^ | χ^2c^ |
| --- | --- | --- | --- | --- |
| GOQDs | 2.08 (0.50) | 0.71 (0.50) | 1.40 | 1.242 |

^a^ Monitored wavelength was 500 nm. The PL decay curves were fitted by a bi-exponential function to calculate the lifetime of sample. ^b^ The intensity weighted average exciton lifetime (*τ*_avr_) was *f*_1_*τ*_1_ + *f*_2_*τ*_2_, where *f*_1_ and *f*_2_ are fractional intensities and *τ*_1_ and *τ*_2_ are lifetimes. ^c^χ^2^ is the reduced chi-square value.
